# Magnetic resonance neurography detects diabetic neuropathy early and with Proximal Predominance

**DOI:** 10.1002/ana.24524

**Published:** 2015-11-14

**Authors:** Mirko Pham, Dimitrios Oikonomou, Benjamin Hornung, Markus Weiler, Sabine Heiland, Philipp Bäumer, Jennifer Kollmer, Peter P. Nawroth, Martin Bendszus

**Affiliations:** ^1^ Department of NeuroradiologyHeidelberg University HospitalHeidelbergGermany; ^2^ Department of Medicine I and Clinical ChemistryHeidelberg University HospitalHeidelbergGermany; ^3^ Department of NeurologyHeidelberg University HospitalHeidelbergGermany; ^4^ Clinical Cooperation Unit Neurooncology, German Cancer Research CenterHeidelbergGermany; ^5^ Section of Experimental RadiologyDepartment of NeuroradiologyHeidelberg University HospitalHeidelbergGermany; ^6^ German Center for Diabetes Research (DZD)

## Abstract

**Objective:**

The aim of this work was to localize and quantify alterations of nerve microstructure in diabetic polyneuropathy (DPN) by magnetic resonance (MR) neurography with large anatomical coverage.

**Methods:**

Patients (N = 25) with mild‐to‐moderate (Neuropathy‐Symptom‐Score [NSS]/Neuropathy Deficit Score [NDS] 3.8 ± 0.3/2.6 ± 0.5) and patients (n = 10) with severe DPN (6.2 ± 0.6/7.4 ± 0.5) were compared to patients (n = 15) with diabetes but no DPN and to age‐/sex‐matched nondiabetic controls (n = 25). All subjects underwent MR neurography with large spatial coverage and high resolution from spinal nerve to ankle level: four slabs per leg, each with 35 axial slices (T2‐ and proton‐density–weighted two dimensional turbo‐spin‐echo sequences; voxel size: 0.4 × 0.3 × 3.5 mm^3^) and a three‐dimensional T2‐weighted sequence to cover spinal nerves and plexus. Nerve segmentation was performed on a total of 280 slices per subject. Nerve lesion voxels were determined independently from operator input by statistical classification against the nondiabetic cohort. At the site with highest lesion‐voxel burden, signal quantification was performed by calculating nerve proton spin density and T2 relaxation time.

**Results:**

Total burden of nerve lesion voxels was significantly increased in DPN (*p* = 0.003) with strong spatial predominance at thigh level, where average lesion voxel load was significantly higher in severe (57 ± 18.4; *p* = 0.0022) and in mild‐to‐moderate DPN (35 ± 4.0; *p* < 0.001) than in controls (18 ± 3.6). Signal quantification at the site of predominant lesion burden (thigh) revealed a significant increase of nerve proton spin density in severe (360 ± 22.9; *p* = 0.043) and in mild‐to‐moderate DPN (365 ± 15.2; *p* = 0.001) versus controls (288 ± 13.4), but not of T2 relaxation time (*p* = 0.49). Nerve proton spin density predicted severity of DPN with an odds ratio of 2.9 (95% confidence interval: 2.4–3.5; *p* < 0.001) per 100 proton spins.

**Interpretation:**

In DPN, the predominant site of microstructural nerve alteration is at the thigh level with a strong proximal‐to‐distal gradient. Nerve proton spin density at the thigh level is a novel quantitative imaging biomarker of early DPN and increases with neuropathy severity. Ann Neurol 2015;78:939–948

Distal symmetrical sensorimotor diabetic polyneuropathy (DPN), also known as length‐dependent diabetic polyneuropathy, is by far the most common type of diabetic neuropathy.^1^, ^2^ The descriptive terms *distal symmetrical* and *length dependent* mainly refer to the spatial distribution and temporal evolution of neuropathic signs and symptoms, which initially involve the very distal regions of the lower extremities. During the early stages of disease, sensory signs and symptoms typically occur first at the tip of the toes and in the feet and are later most severe in these distal regions (eg, loss of sensation across modalities, tingling, burning pain, paresthesia, and numbness). Progression of DPN may be associated with the proximal extension of sensory and/or motor signs and symptoms also involving the ankle or further proximal levels. One important aspect of DPN pathogenesis that remains poorly understood is the spatiotemporal distribution and propagation of microstructural nerve alteration. The most prominent histological fingerprint of DPN is a distally dominant, mixed axonal loss involving all types of nerve fibers.^3^, ^4^, ^5^, ^6^, ^7^, ^8^, ^9^ However, where along the lower extremity peripheral nerves, including their terminal fibers, and the microvascular and/or metabolic pathologic mechanisms by which axon structure degeneration occurs first remain controversial issues. One view holds that early axon degeneration occurs distally.^9^ Accordingly, the term *dying‐back degeneration* has been coined to reflect this assumption that primary/early structural degeneration occurs with distal predominance and propagates over time from distal to more proximal levels. An alternative hypothesis is that early axon loss occurs first and subsequently accumulates at more proximal levels (eg, thigh level), leading to a length‐dependent degeneration by which the most severe fiber loss is also located distally as a downstream consequence.^5^, ^6^ To address this question, we used quantitative in vivo magnetic resonance (MR) neurography, which provides extensive and contiguous coverage of the lower extremity nerves while simultaneously maintaining high structural resolution. A more precise understanding of the location of the primary/early focus of nerve microstructural alteration would not only provide a better knowledge of DPN pathogenesis, but also offer a target location to aid in early prognosis, diagnosis, and, importantly, the early monitoring of the microstructural effects of any therapeutic intervention.

## Materials and Methods

This study was approved by the local ethics board (S‐057), and informed written consent was obtained from all participants. Fifty patients with type 1 or 2 diabetes and 25 age‐/sex‐matched, nondiabetic healthy control subjects were prospectively enrolled using the following exclusion criteria: age < 18 or > 75; any history of symptomatic peripheral artery or cerebrovascular disease, alcoholism, end‐stage renal disease, or any other disease known to be related to the manifestation of peripheral neuropathy (eg, autoimmune disease, systemic vasculitis, or infectious diseases); and any contraindication for magnetic resonance imaging (MRI).

### Clinical and Neurophysiological Determination and Grading of DPN

Motor, sensory, and autonomic symptoms were scored according to the Neuropathy Symptom Score (NSS).^10^ A detailed neurological examination was performed in each patient, including evaluation of the Neuropathy Disability Score (NDS).^10^ Each patient underwent nerve conduction studies (NCS) of the right peroneal, sural, and left tibial nerves by surface electrostimulation at standard sites (distal‐motor latencies, compound‐motor or sensory‐action potentials and nerve‐conduction velocities). Skin temperature was controlled at a minimum of 32 °C, and NCS cut‐off values were adjusted for age. The presence of DPN was confirmed if one or both of the following criteria were fulfilled:

1. A score of ≥ 3 on NDS or NSS. In case of a discrepancy between NDS and NSS, the more severely altered score was selected for classification according to the guidelines of the German Society for Diabetology.
2. Abnormal NCS of two different nerves according to the standard reference values used by our clinical neurophysiology laboratory.

The severity of DPN was classified as either mild to moderate (NDS ≤ 8 or NSS ≤ 6) or severe (NDS > 8 or NSS > 6).

### MRI Data Acquisition

All MRI examinations were performed on a 3‐Tesla MR‐scanner (TRIO, Siemens, Erlangen, Germany). The following extensive examination protocol of pulse sequences was performed separately for each leg and in each participant following the exact same order:

1. Three‐dimensional T2‐weighted inversion recovery SPACE (Sampling Perfection with Application‐optimized Contrasts using different flip angle Evolution) sequence with 50 axial reformations for imaging the lumbosacral plexus and spinal nerves: repetition time/echo time (TR/TE) 3,000/202 ms, field of view (FOV) 305 × 305 mm^2^, matrix size 320 × 320 × 104, and voxel size 1.0 × 1.0 × 1.0 mm^3^.
2. Axial T2‐weighted turbo‐spin‐echo two‐dimensional sequences with spectral fat saturation: TR/TE 5970/55 ms, FOV 150 × 150 mm^2^, matrix size 512 × 512, slice thickness 3.5 mm, interslice gap 0.35 mm, voxel size 0.4 × 0.3 × 3.5 mm^3^, and 35 slices. Slab 1: proximal thigh to mid‐thigh; slab 2: lower leg with alignment of its proximal edge with the tibiofemoral joint; slab 3: ankle level with alignment of its distal edge with the tibiotalar joint (three slabs per leg).
3. Axial dual‐echo turbo‐spin‐echo 2D sequence with spectral fat saturation: TR 5,210 ms, TE_1_/TE_2_ 12/73 ms, FOV 150 × 150 mm^2^, matrix size 512 × 512, slice thickness 3.5 mm, interslice gap 0.35 mm, voxel size 0.4 × 0.3 × 3.5 mm^3^, and 35 slices. One slab per leg: mid‐thigh to distal thigh level (alignment of its distal edge with the tibiofemoral joint).

The net imaging time was 52 minutes 13 seconds (both legs per patient), with coil repositioning requiring an additional 20 to 30 minutes.

### Image and Statistical Analysis

**Figure 1:**
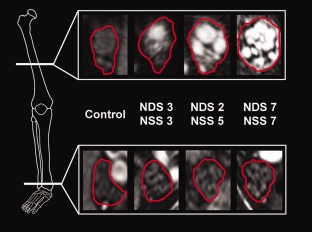
Array of representative source images indicating segmented tibial fascicles within the sciatic nerve at the thigh level (upper row) and their continuation as the tibial nerve distally at the ankle level (lower row). Nerve lesions are conspicuous under hyperintense signal alteration, which was found to be an effect of increased proton spin density by quantitative signal analysis. Within symptomatic diabetic polyneuropathy (DPN) groups (mild/moderate or severe), there is a strong proximal focus of lesion predominance located at the thigh level (the white reference line in the bony anatomical scheme on the left indicates the slice position). A strong proximal‐to‐distal lesion gradient with a marked decrease at the distal ankle level (lower white reference line at the ankle level) and increasing fascicular involvement across groups of increasing DPN severity (from left to right) were observed. NDS = Neuropathy Disability Score; NSS = Neuropathy Symptom Score.

At the far proximal level, the spinal nerves and lumbosacral plexus were covered by pulse sequence 1. For this type of pulse sequence, signal analysis was performed by calculating the contrast‐to‐noise ratios from three regions of interest (ROIs): spinal nerves L5 and S1, each immediately distal to its respective dorsal‐root ganglion, and the lumbosacral plexus, immediately proximal to the infrapiriform foramen. Then, on all images from the proximal thigh to ankle level (pulse sequences 2 and 3), the tibial and peroneal fascicles of the sciatic nerve and their distal continuation as either the tibial or common peroneal nerve were identified and manually segmented on each axial imaging slice (total of 21,000 images in 75 participants, 140 axial image slices per leg, 280 per subject). The readily visible contour between nerve fascicles and the epineurium served as the segmentation border (Fig 1). Slice numbering was from 1 (most proximal at the proximal thigh level) to 140 (most distal at the level of the tibiotalar joint space) for tibial nerve fascicles and their continuation as the tibial nerve. Slice numbering for peroneal nerve fascicles and their continuation as the common peroneal nerve was from 1 (most proximal at the proximal thigh level) to 60 (head of the fibular bone). Branches of the common peroneal nerve distal to the bony fibular head were not reliably recognizable in all subjects and were therefore not included in further analyses. The voxels contained within the ROIs obtained from segmentation are further referred to as *tibial* or *peroneal* nerve voxels.

#### Normalization of nerve voxel signal intensity and binary classification of nerve voxels as lesion voxels

The normalization of nerve voxel signal intensity and operator‐independent automated binary classification of nerve voxels as lesion or nonlesion voxels have been validated and described in detail recently.^11^ Briefly, the histogram distribution of nerve voxel signal intensities (frequency distribution of signal intensities within 100 bins of equal width) was calculated for the age‐/sex‐matched, nondiabetic control population and specifically for each slice position, for each nerve territory (tibial or peroneal origin) and for each side (right or left). Each bin class (for each subject, slice position, nerve territory, and side) was then divided by the bin class containing the central histogram peak of the nondiabetic control population. Consequently, the signal intensity histograms of each subject were normalized by centering around the histogram peak of the control population. Subsequently, nerve voxels that exceeded a binary threshold of normalized signal intensity were classified as nerve lesion voxels and automatically counted. We intentionally made no a priori assumptions regarding a certain cut‐off value. Instead, by iteratively varying the binary thresholds, we found and eventually applied a cut‐off value of > 1.5 (normalized signal intensity) because at this threshold; the differences in nerve lesion voxels were maximized between groups.

#### Proximal‐to‐distal mapping of nerve lesion voxel burden

**Figure 2:**
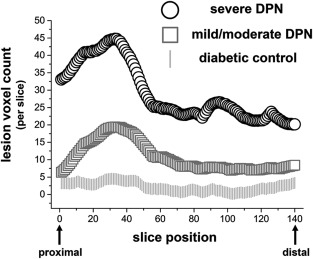
Spatial mapping of lesion voxel count per position from the proximal thigh (slice position 1) to ankle levels (slice position 140). The spatial predominance of the lesion voxel burden in symptomatic diabetic polyneuropathy (DPN; mild/moderate or severe) is located proximally (thigh level slice positions 1–70) and is highest at this location for severe DPN. Increasing symptom severity is associated with an overall increase in the total lesion voxel burden, which is most pronounced proximally (at the thigh level, left side of the *x*‐axis) and less marked distally (at the ankle level, right side of the *x*‐axis). The differences in the proximal lesion count are significant for severe DPN (*p = 0.013) and mild‐to‐moderate DPN (**p = 0.003) compared to nondiabetic controls.

To describe their spatial information and localization, nerve lesion voxels of the tibial nerve territory, including the tibial division of the sciatic nerve and its distal continuation as the tibial nerve, were anatomically mapped from the proximal thigh level (slice number 1) to the ankle level (slice number 140; see Fig 2). To statistically analyze group differences in total nerve lesion voxel burden, one‐way analysis of variance (ANOVA) was performed with four levels of *group* (nondiabetic controls, asymptomatic diabetic patients, mild/moderate DPN, and severe DPN). The association between spatial lesion predominance and symptom severity was tested by repeated‐measures ANOVA with *group* as the between‐subjects factor and *location* as the within‐subjects factor with two levels (proximal vs distal). The *proximal* nerve lesion voxel burden was averaged over slices 21 to 46, corresponding to the region harboring the local lesion maximum (Fig 2), whereas the *distal* nerve lesion voxel burden was averaged over the distal counterpart region (slices 95–120).

### Signal Quantification: T2 Relaxation Time and Proton Spin Density

A higher nerve lesion voxel burden reflects increased nerve voxel signal intensities relative to the normative nondiabetic cohort, but is not a quantitative measure per se. Therefore, quantification of the MR signal by calculating estimates of the two microstructural MR constants (1) apparent nerve T2 relaxation time (Equation (1): T2_app_) and (2) nerve proton spin density (Equation (2): ρ/rho) was performed, which required the acquisition of an additional pulse sequence (pulse sequence 3). Because of the considerable time needed for the acquisition of the extensive imaging protocol for coverage of large anatomical areas and simultaneous high spatial resolution, signal quantification was not possible over the entire area covered and was possible only within this anatomically restricted imaging slab. We chose the thigh level for signal quantification because preliminary measurements in a few patients with DPN and the results from a pilot study^12^ indicated that the lesion burden would be highest at the thigh level and because historical histological data reported proximal nerve lesions at the thigh level.^4^, ^13^ Comprehensive statistical analyses of both quantitative measures were performed by ordered logistic regression on *group*. As additional measures of diagnostic performance, the area under the curve (AUC) from receiver operating characteristic analysis and sensitivity and specificity values were determined

(1) $$T2app=\frac{\left(TE2-TE1\right)}{\ln\left(\frac{SI_{\text{TE1}}}{SI_{\text{TE2}}}\right)}$$

(2) $$\text{ρ}=\frac{SI_{\text{TE1}}}{\exp\left(-\frac{TE1}{T2app}\right)}$$

### Quantification of Nerve Caliber

Nerve caliber was quantified as the total volume of tibial/peroneal voxels per slice. The differences in the mean nerve caliber between groups and between locations (proximal vs distal) and the interaction between group and location were tested by repeated measures ANOVA.

### Comprehensive Analysis of Clinical Variables of Interest

Among patients with diabetes, any potential association between clinical variables of interest and ρ was analyzed by step‐wise backward multiple regression and included the following: age; duration of diabetes, glycosylated hemoglobin (HbA_1c_) level, NSS, NDS, type of diabetes (type I or II), symptomatic DPN, retinopathy, nephropathy, hyperlipidemia, coronary heart disease (CHD), smoking, body mass index (BMI), and body weight.

## Results

### Clinical and Electrophysiological Data

Detailed demographic, clinical, and electrophysiological data for the three patient groups are given in Table 1. In the first group (patients with diabetes but no symptoms or signs of DPN), the mean NSS and NDS were 0.9 ± 0.2/0.1 ± 0.1. In the second group (mild/moderate DPN), the NSS/NDS were 3.8 ± 0.3/2.6 ± 0.5. In the third group (severe DPN), the NSS/NDS were 6.2 ± 0.6/7.4 ± 0.5. Age‐/sex‐matched, nondiabetic subjects served as controls (N = 25; mean age: 56.4 ± 1.5 years; 13 males/12 females).

**Table 1:** Demographic, Clinical, and Electrophysiological Data of Different Patient Groups

	Diabetes No DPN	DPN Mild or Moderate	DPN Severe
N	15	25	10
Age	59.1 ± 2.3	64.0 ± 1.5	66.9 ± 2.6^a^
Sex (M/F)	9/6	16/9	7/3
DM duration (yr)	28.7 ± 3.6	23 ± 2.8	17.9 ± 3.6
DM type I/II	10/5	10/15	8/2
NSS	0.9 ± 0.2	3.8 ± 0.3^b^	6.2 ± 0.6^b^
NDS	0.1 ± 0.1	2.6 ± 0.5^b^	7.4 ± 0.5^b^
Retinopathy	26.7%	32%	40%
Nephropathy	7%	32%	30%
CHD	7%	20%	20%
Hypertension	67%	92%	80%
Hyperlipidemia	53%	84%	80%
Smoking	27%	8%	20%
HbA_1c_ (%)	7.73 ± 0.29	7.45 ± 0.28	7.79 ± 0.55
Cholesterol (mmol/L)	177.4 ± 11.5	189.6 ± 12.2	177.9 ± 9.9
Triglycerides (mmol/L)	117.8 ± 20.7	236.5 ± 47.3	158.0 ± 38.9
HDL (mmol/L)	58.0 ± 4.5	60.5 ± 4.7	57.0 ± 8.4
eGFR (ml/min/L)	91.9 ± 3.8	82.3 ± 3.7	75.4 ± 4.2^a^
BMI (kg/m^2^)	27.4 ± 1.0	28.7 ± 0.9	30.2 ± 1.2
Sural NCV (m/s)	43.6 ± 1.6	40.4 ± 2.2	35.5 ± 4.2^a^
Sural SNAP (μV)	9.7 ± 1.9	3.6 ± 0.9^b^	2.4 ± 1.2^b^
Tibial NCV (m/s)	43.4 ± 1.3	44.0 ± 1.1	38.4 ± 2.7
Tibial CMAP (mV)	14.8 ± 1.8	9.3 ± 1.1^a^	9.5 ± 2.5
Peroneal NCV (m/s)	43 ± 1.2	42.3 ± 1.0	39.2 ± 1.6
Peroneal CMAP (mV)	6.3 ± 0.8	4.1 ± 0.7^a^	3.3 ± 0.9^a^

All values are expressed as the mean ± standard error, median, or percentage. Values of the symptomatic groups (middle and right columns) were tested pairwise against the corresponding values in the patient group without symptoms (Diabetes ‐ no DPN, left).

Statistical significance at ^a^ *p* < 0.05 and ^b^ *p* < 0.01.

DM = diabetes mellitus; NSS = Neuropathy Symptom Score; NDS = Neuropathy Disability Score; CHD = coronary heart disease; HbA_1c_ = glycosylated hemoglobin; HDL = high‐density lipoprotein; eGFR = estimated glomerular filtration rate; BMI = body mass index; NCV = nerve conduction velocity; SNAP = sensory nerve action potential; CMAP = compound motor action potential; DPN = diabetic polyneuropathy.

### Nerve Lesion Voxel Mapping

Figure 1 displays representative high‐resolution MR neurography source images at the thigh level, which was found to be the anatomical site of the highest lesion voxel burden in the symptomatic groups. Proximal source images at the thigh level are compared with distal source images at the ankle level, where a significantly lower lesion voxel burden was observed. These high‐resolution images demonstrate that nerve lesion voxels form clusters that correspond to nerve fascicles. The lesion voxel counts per slice position are plotted in Figure 2 from the proximal thigh (slice 1) to ankle level (slice 140). The spatial predominance of increased lesion voxel count was located at the thigh level in both groups of symptomatic DPN (severe and mild/moderate). The mean total *tibial* lesion voxel count was 4.4 × 10^3^ for severe DPN, 1.7 × 10^3^ for mild/moderate DPN, and 0.28 × 10^3^ for patients with diabetes but no symptoms of DPN. The mean total *peroneal* lesion voxel count was 1.7 × 10^3^ for severe DPN, 1.1 × 10^3^ for mild/moderate DPN, and 0.26 × 10^3^ for patients with diabetes but no symptoms of DPN. Statistical analysis by one‐way ANOVA showed significant differences in the mean total burden of tibial and peroneal lesion voxels between groups (*F* = 5.4_(3,71)_; *p* = 0.0021). Post‐hoc pair‐wise comparisons showed that the lesion burden in severe DPN was significantly higher than in nondiabetic controls (*p* = 0.003, corrected) and was also higher in mild/moderate DPN than in nondiabetic controls (*p* = 0.10, corrected).

Repeated‐measures ANOVA confirmed the significant main effect of *group* on the total lesion burden (*F* = 6.3_(3,71)_; *p* = 0.0008) and revealed a significant main effect of *location* (*F* = 72.7_(1,74)_; *p* < 0.0001) and a significant interaction of *group × location* (*F* = 5.2_(3,71)_; *p* = 0.0027). The interaction *group × location* indicates that the effect of symptom severity on lesion burden depended on the anatomical position. Post‐hoc contrasts showed the direction of this interaction. The mean lesion voxels per *location*, corresponding standard errors and test statistics are given in Table 2. *p* values were adjusted to account for multiple comparisons with the Bonferroni‐Holm correction. Table 2 shows that the mean lesion voxel count was significantly higher at the proximal location (thigh) for each of the two symptomatic groups (mild/moderate and severe DPN), but not for asymptomatic patients with diabetes compared with non‐diabetic controls.

**Table 2:** Mean Lesion Voxel Counts Per Slice at Proximal and Distal Locations for Each Group

	Control (nondiabetic)	Diabetic Control (no DPN)	Mild/Moderate DPN	Severe DPN
Proximal lesion count	18 ± 3.6	21 ± 5.5	35 ± 4.0	57 ± 18.4
Test statistics (vs controls)		*F*(1,38) = 0.73; *p* = 0.3977	*F*(1,48) = 13.95; *p* = 0.0005	*F*(1,33) = 10.99; *p* = 0.0022
Distal lesion count	8 ± 1.4	8 ± 2.9	12 ± 1.8	22 ± 8.1
Test statistics (vs controls)		*F*(1,38) = 0.01; *p* = 0.9369	*F*(1,48) = 2.22; *p* = 0.1447	*F*(1,33) = 6.27; *p* = 0.0174

Post‐hoc pair‐wise comparisons were performed for each group and location against the age‐/sex‐matched nondiabetic control group.

DPN = diabetic polyneuropathy.

At the far proximal spinal nerve and plexus levels, no differences were observed between groups in any of the three evaluated ROIs: spinal nerve L5 (*p* = 0.21), S1 (*p* = 0.31), and lumbosacral plexus (*p* = 0.13), immediately proximal to the infrapiriform foramen.

### Signal Quantification at the Site of Predominant Lesion Burden

**Figure 3:**
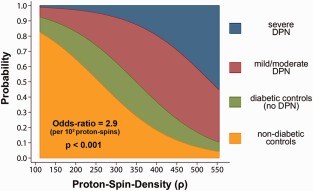
Ordered logistic regression analysis was significant for the quantitative microstructural marker of proton spin density (ρ/rho). This plot illustrates the cumulative probabilities predicted from the logistic regression model for different clinical stages of diabetic polyneuropathy (DPN) as functions of quantitated proton spin density (*x*‐axis). An increase of 10^2^ proton spins was significantly associated (*p* < 0.001) with an odds ratio of 2.9 (95% confidence interval: 2.4–3.5) for predicting higher symptom severity.

Ordered logistic regression revealed a significant effect of ρ (*p* < 0.001), but not of T2_app_ (*p* = 0.493). The odds ratio to present with higher symptom severity was 2.9 (95% confidence interval [CI]: 2.4–3.5) per 10^2^ proton spins (ρ). The mean proton‐spin densities were 360 ± 22.9 for severe DPN (corrected *p* = 0.043 vs nondiabetic controls), 365 ± 15.2 for mild/moderate DPN (corrected *p* = 0.001 vs nondiabetic controls), and 323 ± 14.4 for patients with diabetes but no symptoms or signs of DPN (corrected *p* = 0.57 vs 288 ± 13.44 nondiabetic controls). In Figure 3, the predicted cumulative probabilities of increasing symptom severity are plotted as a function of ρ.

The diagnostic accuracy of ρ at the thigh level to discriminate symptomatic DPN (mild/moderate or severe) from nondiabetic controls was AUC = 0.77 (95% CI: 0.65–0.89). Sensitivity/specificity values were 83%/52% at a threshold of ≥294 (69%/72% at ≥ 336 and 57%/88% at ≥ 357). The same accuracy was found when only the discrimination between mild/moderate DPN and controls was analyzed (AUC = 0.77).

### Quantification of Nerve Caliber

A significant increase in the nerve caliber was observed between groups (*F* = 5.61_(3,71)_; *p* = 0.001) with increasing symptom severity (tibial/peroneal: controls 52.8 ± 1.4/23.5 ± 1.1 mm^3^, diabetes without DPN 60.4 ± 3.3/23.6 ± 1.2, mild/moderate DPN 62.5 ± 2.7/24.6 ± 1.4, and severe DPN 74.4 ± 6.0/29.2 ± 3.0). This caliber increase between groups occurred regardless of proximal or distal location, that is, the caliber increase with increasing symptom severity was equally observed at both the proximal and distal levels (interaction *group* × *location: F* = 1.16_(3,71)_; *p* = 0.33). Within each group, the nerve caliber significantly decreased from the proximal to distal levels, as would be expected because of the natural anatomical reduction in size along the trunks of peripheral nerves (*F* = 113.14_(1,74)_; *p* < 0.0001).

### Correlation of ρ With Clinical Variables of Interest

The only significant clinical predictors of ρ were the presence of symptomatic DPN (*p* = 0.032; regression coefficient = 71.25; 95% CI: 6.47–136.03), NDS (correlation coefficient = 0.3; *p* = 0.009), and NSS (correlation coefficient = 0.27; *p* = 0.02). All other clinical variables, that is, the type of diabetes (*p* = 0.194), HbA_1c_ level (*p* = 0.993), hyperlipidemia (*p* = 0.991), duration of diabetes (*p* = 0.980), smoking (*p* = 0.925), the presence of nephropathy (*p* = 0.218) or retinopathy (*p* = 0.632), body weight (*p* = 0.83) and BMI (*p* = 0.81), were not significantly associated with ρ.

## Discussion

One important limitation in diagnosing and monitoring DPN during the early stages is the inability to directly and noninvasively detect alterations in the nerve microstructure in the lower extremities in vivo. In the lower extremity peripheral nervous system (PNS), where the symptoms of DPN occur first and continue to dominate during the clinical course of the disease, alterations in nerve microstructure could be observed only ex vivo in very small, restricted fractions of nerve tissue (mostly from sural nerve and skin biopsies) or by NCS, methods that reflect structural changes only indirectly by measuring electrophysiological function. Indeed, it has been estimated that less than 10% of the total nerve fiber population contributes to the signal recorded on the skin surface by NCS.^14^

In the past, efforts to understand the spatiotemporal sequence of axon degeneration or of any other suspected primary/early event of structural alteration were limited to making inferences based on histopathological observations ex vivo. However, where along the lower extremity nerves the potentially inciting metabolic and/or vascular events occur first and how the consequent microstructural alteration propagates in space over time remains to be demonstrated in vivo.

It is generally agreed that fiber loss in lower extremity peripheral nerves is the most prominent histological fingerprint of DPN and involves large myelinated and/or small fibers.^3^, ^4^, ^5^, ^6^, ^7^, ^8^, ^9^ In accord with the distal dominance of symptoms and signs, the longitudinal gradient of fiber loss along lower extremity nerve trunks is also directed from distal (where it is most severe) to proximal.^4^, ^5^, ^6^, ^7^, ^9^ Such a distally marked reduction in fiber density appears not only in sural nerve biopsies, but also in skin punch biopsies, which reveal the density of cutaneous innervation by small fibers (intraepidermal nerve fiber density; IENF). In healthy subjects, IENF is substantially higher at the thigh level than at the ankle level. In DPN, IENF is reduced at both the distal and proximal levels, but again, this decrease is most obvious distally.^15^, ^16^

However, the spatiotemporal progression of fiber loss and the events that trigger fiber loss remain unclear. One view holds that axons degenerate “centripetally” or in a “dying‐back” fashion (ie, from distal to more proximal levels).^9^, ^17^ This concept is mainly based on fascicular biopsies taken from distal sural nerves at the ankle level. Through very extensive and meticulous histological analyses, a few studies have reported results for the distal levels of the sural, peroneal, and tibial nerves and, importantly, also for defined proximal levels, including several serial sections of spinal nerves, the plexus, and the sciatic nerve at the thigh level.^4^, ^6^, ^13^ In these studies, Dyck et al,^4^ Sugimura and Dyck,^13^ and Johnson et al^6^ consistently showed that fiber loss at more proximal levels (eg, in the sciatic nerve at the thigh level) occurs not diffusely, but instead within focal and multifocal zones that appear “punched out” from the sciatic nerve fascicles, increasing in severity at more distal levels. This focal/multifocal lesion pattern on sciatic nerve cross‐sections at the thigh level closely resembled the focal/multifocal fiber loss observed after experimentally induced ischemic insults, for example, by microsphere embolization into vessels supplying the sciatic nerve^18^ or in human necrotizing vasculitic neuropathy.^19^ Based on these findings, Dyck et al were among the first to make assumptions regarding the temporal course and location of events in the pathogenesis of DPN. These investigators suggested that structural alterations of the interstitium and/or nerve microcirculation, possibly in combination with metabolic mechanisms, begin proximally, accumulate from proximal to distal, and eventually lead to length‐dependent fiber loss at more distal levels.^4^, ^5^ However, this suggestion has not been developed further during the past three decades and remains controversial because it was based on ex vivo observations at a single point in time by nerve biopsy or postmortem sampling.

Here, we used quantitative MR neurography for high‐resolution in vivo imaging of the lower extremity PNS with very large and contiguous anatomical coverage. We observed a clear proximal‐to‐distal gradient of nerve lesions with proximal lesion predominance at the thigh level. These imaging findings differentiate the nerve lesion gradient from the longitudinal gradient of symptoms in DPN, which typically follow the opposite direction (strong distal focus). Interestingly, nerve lesion voxels formed clusters corresponding to nerve fascicles and exhibited focal/multifocal distributions at the thigh level (Fig 1) that showed striking similarity to the historical histopathological observations in this region.^4^, ^6^, ^13^ The extensive anatomical coverage allowed us to map the burden of nerve lesion voxels from far proximal to ankle levels. With this approach, a nondiffuse, but focal, pattern of nerve signal increase could be localized to the thigh level (Figs 1 and 2). Furthermore, we were able to perform additional MR measurements at the thigh level to quantitatively determine which microstructural alteration parameters were responsible for the observed proximal signal increase in symptomatic DPN patients. Extending the quantitative signal analysis to distal levels was impossible because of the long duration of the imaging protocol.

Thus, the significant increase in the proximal nerve signal was found to be an effect of increased proton‐spin density (ρ), but not of altered T2 relaxation time. Increased ρ at the thigh level was a significant predictor of symptomatic DPN (Fig 3) and was detectable not only in advanced stages (mean NDS/NSS 6.2/7.4 in the group with severe DPN), but even in early DPN of mild/moderate severity (mean NDS/NSS scores of 3.8/2.6). These results suggest that ρ at the thigh level as quantified by MR neurography is a novel, noninvasive, and clinically meaningful surrogate measure of diabetic microstructural nerve alteration in vivo. We must emphasize that our investigation was not designed as a diagnostic study. Therefore, head‐to‐head comparisons of diagnostic accuracy for the early detection of DPN with established methods, such as electrophysiology or skin biopsy, should be performed in the future.

The cross‐sectional design of our study limits any interpretation regarding the temporal course of events. However, the significant increase in nerve lesions with DPN progression, which was represented in this study by cross‐sectional cohorts with different stages of DPN, showed greater differences proximally (at the thigh level) than distally. This finding provided in vivo evidence of the controversial concept that proximal alteration of the nerve structure may be an early/primary event that accumulates and eventually causes length‐dependent fiber loss. Similarly, in familial amyloid polyneuropathy (FAP), which exhibits a clinical and electrophysiological phenotype that shares many features of DPN, proximal rather than distal alteration of the nerve microstructure was observed recently using the same imaging methods.^11^ Despite the uniform location of the proximal nerve signal increase at the thigh level in both DPN and FAP, important distinctions can now be made between these two different etiologies based on the quantifiable microstructural markers of ρ and T2: In DPN, symptomatic manifestation is reflected by increasing ρ, as reported here, whereas in FAP, symptomatic manifestation was previously reported to be strongly linked to an increase in T2.^11^ Furthermore, nerve fascicle imaging in FAP revealed an entirely diffuse pattern of proximal nerve fascicle signal increase [Fig 4 in Kollmer et al^11^], instead of the focal/multifocal pattern observed here in DPN. These differences between FAP and DPN observed in‐vivo by MR neurography are in close agreement with similar histopathological differences between DPN^4^, ^5^, ^6^, ^13^ and FAP. In FAP focal/multifocal patterns of fiber loss have never been reported.

The dissociation between the proximal predominance of nerve microstructure alteration as observed by imaging and the typical predominance of symptoms at the distal level, where fiber loss is known to be the prominent histological finding, clearly indicates that the imaging methods used here allow for the observation of aspects of microstructural pathology other than fiber loss. Because biopsies of sciatic, peroneal, and tibial nerve trunks at the thigh level are not possible for scientific purposes, any interpretation of our imaging data cannot be based on direct correlation with histology, but instead must rely on (1) historical histological specimens of distal and proximal nerve segments,^4^, ^5^, ^6^, ^13^ as noted above, and (2) the following physical considerations regarding how the MR signal is influenced by microstructural sources on a quantitative level.

Unlike nerve proton spin density, the nerve‐apparent T2 relaxation time did not contribute significantly to the elevated MR signal in our study. This evidence indicates that the free hydrogen protons of extracellular water (ie, endoneurial edema) did not contribute substantially to the observed signal increase. An increase in free water protons resulting from endoneurial edema would be expected to constitute a strong driving force increasing T2_app_, but not nerve proton spin density, ρ.^20^, ^21^ The fact that nerve lesions on imaging were an effect of increased ρ and not of altered T2_app_ suggests a change in the macromolecular organization of the extracellular compartment.^22^ In fact, several distinct pathogenic metabolic pathways,^23^ among which the formation of advanced glycation endproducts (AGEs) from reactive metabolites is one of the most intensively studied, have been linked to functional^24^ and structural^25^, ^26^, ^27^ compromises in the extracellular macromolecular environment. A variety of specific molecular and cellular consequences of AGE and other pathogenic pathways could be identified, all of which could be linked to similar structural effects: the overproduction of extracellular matrix proteins^25^, ^26^, ^27^, ^28^ and a leakage of plasma proteins through the blood–nerve barrier.^29^ Endoneurial fibrosis,^25^, ^26^, ^27^, ^28^ the pathogenesis of a proinflammatory milieu,^30^, ^31^ and microvascular dysfunction/occlusions^32^ are commonly considered to be the final structural consequences of these processes.^23^ All of these microstructural alterations can clearly be expected to elevate ρ, as quantitatively observed here through imaging.

In summary, we identified a novel, noninvasive, and quantifiable imaging biomarker able to localize and quantify microstructural nerve alteration in DPN in vivo. This marker, ρ, was closely associated with clinical symptoms and was significantly elevated in mild/moderate DPN (low mean NDS/NSS scores of 3.8/2.6). By determining the microstructural nerve alteration over a very large anatomical area, we identified a proximal‐to‐distal lesion gradient, which is in close agreement with historical findings ex vivo.^4^ This gradient constitutes in vivo evidence that microstructural nerve alteration in DPN may not diffusely extend along the entire nerve length, but may instead occur with spatial predominance at the thigh level. Why nerve segments at the thigh level should be susceptible to microstructural alteration induced by ischemic and/or metabolic mechanisms is unknown. It can be speculated that these nerve segments experience inferior protection by nutritive microvascular supply because they are located at a hemodynamic watershed region of nerve perfusion.^33^, ^34^, ^35^ Altogether, the accumulation of microstructural nerve alterations at the thigh level, which may represent a vulnerable region for ischemic and/or metabolic injury, may precede and possibly trigger distal fiber loss in a length‐dependent manner in which the distal fibers tend to die first.

Our results suggest that future efforts related to the early detection and risk prediction of DPN should also probe the thigh level as the anatomical site where early structural nerve injury may predominate. The novel DPN imaging biomarker reported on here may also facilitate the earlier detection of DPN and the monitoring of the beneficial structural effects of therapeutic substances in intervention trials and may therefore contribute to reducing the cost of discovering efficient therapies to prevent or reverse DPN, which remain entirely lacking.

## Authorship

M.P., D.O., P.B., S.H., P.N., and M.B. conceived and designed the study. Acquisition and analysis of imaging data including segmentation procedures were accomplished by B.H., J.K., and M.P. Acquisition and analysis of clinical and electrophysiological data were accomplished by D.O., M.W., and M.P. M.P., D.O., P.N., and M.B. were responsible for writing and drafting the manuscript.

## Potential Conflicts of Interest

This study was funded, in part, by the EFSD/JDRF/Novo Nordisk European Programme in Type 1 Diabetes Research (M.P.). Novo Nordisk manufactures and markets pharmaceutical products for the treatment of diabetes.
